# Plasma Lipidomic Signature of Rectal Adenocarcinoma Reveals Potential Biomarkers

**DOI:** 10.3389/fonc.2017.00325

**Published:** 2018-01-08

**Authors:** Márcia Cristina Fernandes Messias, Giovana Colozza Mecatti, Célio Fernando Figueiredo Angolini, Marcos Nogueira Eberlin, Laura Credidio, Carlos Augusto Real Martinez, Cláudio Saddy Rodrigues Coy, Patrícia de Oliveira Carvalho

**Affiliations:** ^1^Laboratory of Multidisciplinary Research, São Francisco University (USF), Bragança Paulista, São Paulo, Brazil; ^2^Institute of Chemistry, University of Campinas (UNICAMP), Campinas, São Paulo, Brazil; ^3^Department of Surgery, University of Campinas (UNICAMP), Campinas, São Paulo, Brazil

**Keywords:** lipidomic, rectal adenocarcinoma, biomarkers, lipoperoxidation, mass spectrometry

## Abstract

**Background:**

Rectal adenocarcinoma (RAC) is a common malignant tumor of the digestive tract and survival is highly dependent upon stage of disease at diagnosis. Lipidomic strategy can be used to identify potential biomarkers for establishing early diagnosis or therapeutic programs for RAC.

**Objective:**

To evaluate the lipoperoxidation biomarkers and lipidomic signature in the plasma of patients with RAC (*n* = 23) and healthy controls (*n* = 18).

**Methods:**

Lipoperoxidation was evaluated based on malondialdehyde (MDA) and F_2_-isoprostane levels and the lipidomic profile obtained by gas chromatography and high resolution mass spectrometry (ESI-q-TOF) associated with a multivariate statistical technique.

**Results:**

The most abundant ions identified in the RAC patients were those of protonated phosphatidylcholine and phosphatidylethanolamine. It was found that a lisophosphatidylcholine (LPC) plasmalogen containing palmitoleic acid [LPC (P-16:1)], with highest variable importance projection score, showed a tendency to be lower in the cancer patients. A reduction of *n −* 3 polyunsaturated fatty acids was observed in the plasma of these patients. MDA levels were higher in patients with advanced cancer (stages III/IV) than in the early stages groups and the healthy group (*p* < 0.05). No differences in F_2_-isoprostane levels were observed among these groups.

**Conclusion:**

This study shows that the reduction in plasma levels of LPC plasmalogens associated with an increase in MDA levels may indicate increased oxidative stress in these patients and identify the metabolite LPC (P-16:1) as a putatively novel lipid signature for RAC.

## Introduction

Colorectal cancer (CRC) is the third commonest malignant tumor worldwide. Countries with the highest incidence rates are Australia, New Zealand, Canada, the United States, and parts of Europe (1). In Brazil, colon and rectal cancers are among the five most diagnosed cancers and are the third highest cause of cancer-related deaths (2, 3). Rectal cancer is a commonly frequent type of CRC and rectal adenocarcinoma (RAC) is the primary histological type of rectal cancer, associated with considerable mortality and morbidity. The overall 5-year survival rate is described as 66.5% (4).

Important cellular functions, such as signal transduction, post-translational modifications, homeostasis, adhesion, migration, apoptosis, and neurotransmission are regulated by phospholipids (PLs) (5, 6). These molecules are subject to extensive modification in cancer, often with marked alterations in the phosphatidylcholine (PC) and phosphatidylethanolamine (PE) metabolism due to changes in the activity of degradative enzymes, including phospholipase A (7, 8) and anabolic enzymes, especially fatty acid synthase, stearoyl-CoA desaturase (SCD) and choline kinase α (9, 10). Both phospholipase and fatty acid synthase are essential for tumor progression and have been identified as potential cancer treatment targets (11, 12).

Understanding the occurrence of alterations in plasma metabolic profiles and lipid peroxidation associated with tumor onset and progression could lead to better diagnostic tests and could uncover new approaches to prevent or even treat CRC. Decreased serum levels of unsaturated free fatty acids, C16:1, C18:2, C20:4, and C22:6, have been proposed as diagnostic indicators of early stages CRC (13). Also, the risk of CRC is increased with elevations in serum *n −* 3 polyunsaturated fatty acids (PUFA) and *n −* 6 PUFA (14) and with higher levels of plasma saturated fatty acids (SFAs) in plasma PLs, in particular palmitic acid (15). It has been reported that the shift from lipid uptake to *de novo* lipogenesis in cancer cells leads to increased membrane lipid saturation and higher levels of saturated and monounsaturated PLs, thus rendering the cancer cells more protected against the oxidative damage by reducing lipid peroxidation (16). However, inhibition of fatty acid desaturation following ablation of SCD causes oxidative stress, cell cycle inhibition, and apoptosis in cancer cells (17).

Most of the research groups investigating lipidomic profile in CRC to elucidate the changes in lipid metabolism have assessed the tissue (18–22) and exosomes (23). Biofluid-based detection strategies (urine and peripheral blood and its components) are an attractive approach for screening, mainly due to their offer of non-invasive access to large quantities of samples and ease of acquisition. Molecular analyses of early stages rectal cancer-related plasma represent an attractive choice for the discovery and validation of diagnostic biomarkers.

In this study, lipoperoxidation biomarkers [malondialdehyde (MDA) and F_2_-isoprostane] and lipid signatures obtained by gas chromatography (GC) and high-resolution mass spectrometry (ESI-qToF-MS) followed by multivariate data analysis, including principal component analysis (PCA) and (orthogonal) partial least squared discriminant analysis [(O)PLS-DA] were applied for the rapid investigation of potential diagnostic biomarkers in the plasma of RAC patients.

## Experiment

### Participants and Samples

Plasma samples were obtained from 18 healthy volunteers and 23 RAC patients stratified into three groups [stage 0, stages I/II, and stages III/IV according to the TNM classification system of the American Joint Committee on Cancers (AJCC) (24)]. The ethics committee of the São Francisco University (CAAE: 51356315.5.0000.5514) and the Faculty of Medical Sciences of the State University of Campinas (CAAE: 08287012.0.0000.5404) approved the project. Samples and clinical information on the RAC patients were obtained from the Gastrocentro Colorectal Cancer Medical Outpatient Facility of the Faculty of Medical Sciences of the University of Campinas (UNICAMP) in the period 2013 and 2014. All patients over 18 years old with RAC diagnosed by histopathology were included in the study. Data were collected considering the follow information: age, sex, BMI, race, smoker, diagnosis, date of diagnosis (date of biopsy), TNM stage, type of surgical treatment, neo/adjuvant treatment, recurrence, death, and date of death. The samples were taken prior to the patients’ being submitted to surgical procedures, chemical therapy, and/or radiotherapy. Clinical examination coupled with hemato-biochemical analysis was used to screen healthy controls. Informed consent was acquired from participants prior to blood collection. The same protocol was used in the collection of plasma samples from patients and controls. From each subject, 4 ml of whole blood was collected into a tube containing potassium-EDTA. The plasma was promptly separated and stored at −80°C for posterior analysis.

### Lipid Extraction

Lipids were extracted from the plasma (0.8 ml) by the procedure of Folch et al. (25) with 5.0 ml of chloroform–methanol (2:1) and 0.5 ml aqueous solution of 0.1 N NaCl. The lower lipid phase was collected, separated into two fractions for subsequent analyses, and dried under nitrogen.

### Lipid Classes Analysis by Electrospray Ionization Time-of-Flight Mass Spectrometry (ESI-qToF-MS)

The lipid extracts were diluted in 300 µl of methanol:chloroform (2:1) and 100 µl of this solution were re-diluted in 400 µl of acetonitrile:chloroform (3:1). Then 1 µl was injected into an LC (Agilent 1290) injector of an LC-MS system using no column and with a flow of 0.5 ml min^−1^ of acetonitrile:H_2_O (1:1). The mass spectrometry experiments were performed on a 6550 iFunnel q-ToF (Agilent Technologies) coupled with a Dual Agilent Jet Stream ESI source (Dual-AJS-ESI). The positive ion mode was used for the collection of the mass spectra, applying the following conditions: Gas temp. at 290°C, Drying Gas flow at 11 l min^−1^, Nebulizer at 45 psi, Sheath gas temp. at 350°C, Sheath gas flow 12 l min^−1^, VCap 3000, Nozzle voltage 320 V, Fragmenter 100 V, and OCT 1 RFV pp 750 V. Agilent Mass Hunter Qualitative Analysis software version B.07.00 was used to acquire and process the data. The ESI(+)-MS data were exported in CSV files and statistical analyses were performed using MetaboAnalyst 2.0.

### Fatty Acid Analysis by GC

The dry extract of total lipids was subjected to derivatization before GC analysis. The extracts were converted into fatty acid methyl esters using BF_3_ methanol (26) and analyzed by GC using a Chrompack chromatographer (model CP 9001; Chrom Tech, Inc.) with a flame ionization detector and a CP-Sil 88 capillary column (Chrompak, WCOT Fused Silica) as previously reported (27). Fatty acid (FA) identification was made by comparing retention times with authentic standards (Sigma) injected under the same conditions. FA composition, represented as a percentage of total FA, was calculated using area of the chromatogram (Software N2000, G-Chrom).

### Lipoperoxidation Biomarkers

Plasma level of MDA was measured according to the method of Ohkawa et al. (28). MDA level was determined by thiobarbituric acid reactive substances based on the reaction between MDA and thiobarbituric acid (TBA). 250 µl of plasma were mixed with 25 µl of 4% butylated hydroxytoluene in methanol, 1 ml 12% trichloroacetic acid, 1 ml of 0.73% TBA, and 750 µl of 0.1 mol/l Tris–HCl buffer containing 0.1 mmol/l EDTA pH 7.4. The reaction yields a pink MDA-TBA adduct, which was measured by the spectrophotometer at 532 nm. The method was standardized with 1,1,3,3-tetramethoxypropane (malondialdehyde bis dimethyl acetal) and MDA concentration was calculated as nmol/ml.

Free 8-isoprostane (8-epi-PGF_2_) was measured by using an enzyme immunoassay kit (8-isoprostane EIA *Kit*, Cayman Chemical Co., Ann Arbor, MI, USA). The plate was incubated for 90 min at room temperature, the absorbance read at 412 nm, and 8-isoprostane concentration was calculated as pg/ml.

### Statistical and Multivariate Data Analysis

Comparative statistical analysis was conducted to compare the two experimental groups. The Tukey test was applied to the means and standard deviations of FA composition and lipoperoxidation biomarkers, adopting a significance level of *p* < 0.05 (5%). Data assessment used InStat GraphPad 2.0 software, and the graphs were elaborated using OriginLab Origin 8.1 software. To enable the assessment of lipid classes, Agilent Mass Hunter Qualitative Analysis B.07.00 software was used for data acquisition and control. Exporting files in CSV was carried out for the ESI (+)-MS. For the statistical analysis, each molecular feature (ion) was normalized by sum, unsupervised segregation was evaluated using statistical web platform Metaboanalyst 2.0. PCA was performed using pareto and the results were used to show the lipids that most strongly influence the discrimination between groups. To enhance data discrimination, the data were also analyzed using the (orthogonal) partial least squared discriminant analysis [(O)PLS-DA] method. Biomarkers were selected according to their variable importance in projection (VIP) values. In addition, an independent *t*-test (*p* ≤ 0.05) was used to evaluate whether different biomarker candidates were statistically significant between groups. The differences of FA composition between groups were analyzed by one-way ANOVA, followed by the Tukey test and *p* < 0.05 was considered to be statistically significant.

## Results

### Patient Characteristics

A total of 23 patients with RAC and 18 healthy volunteers were included in our study. RAC patients included 4 patients with stage 0 (carcinoma *in situ*) (17.4%), 9 (39.1%) with early stages (TNM staging I/II), and 10 (43.5%) with advanced stages (TNM staging III/IV), between 24 and 91 years old. Table 1 summarizes the major characteristics of all subjects.

**Table 1:** Demographic data and major clinical characteristics of rectal adenocarcinoma (RAC) patients and healthy volunteers.

	RAC patients	Healthy volunteers
*N*	23	18
Sex (M/F)	10:13	10:8
Age (years)	56.6 ± 11.0	55.9 ± 11.9
Race (%)	
White	20 (87)	16 (89)
Black	2 (8.7)	2 (11)
Mulatto	1 (4.3)	0
Yellow^a^	0	0
BMI (kg/m^2^)	27.5 ± 4.6	25.3 ± 5.1
Smoker (%)		–
Yes	4 (17.4)	–
No	8 (34.8)	–
Ex	5 (21.7)	–
No information	6 (26.1)	–
Stages (%)		–
0	4 (17.4)	–
I and II	9 (39.1)	–
III and IV	10 (43.5)	–

*^a^Brazilian census category of Chinese or Japanese descent*.

### Profile Analysis of Lipids in Plasma RAC Patients vs Healthy Volunteers

**Figure 1:**
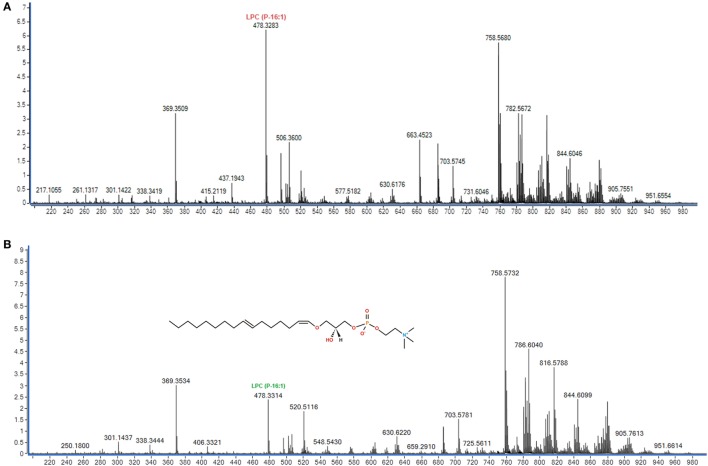
Representative (+) ESI-MS (q-ToF) mass spectra of plasma from **(A)** a healthy volunteer and **(B)** a rectal adenocarcinoma patient. **(B)** Representation of the molecular structure (C_24_H_48_NO_6_P) *m/z* 478 identified as lisophosphatidylcholine [LPC (P-16:1)].

**Figure 2:**
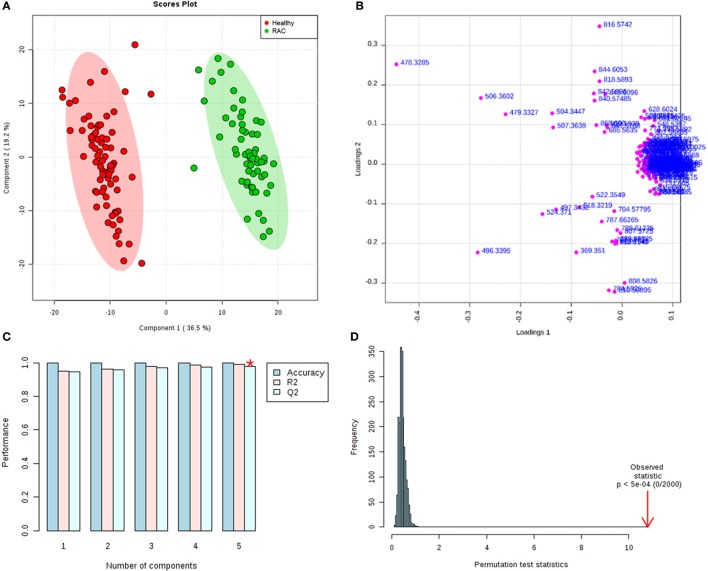
**(A)** PLS-DA scores plot of healthy volunteers (red) and rectal adenocarcinoma patients (green). 36.5 and 19.2% are the scores of the component 1 and 2, respectively. **(B)** Loadings plot for components 1 and 2. **(C)** Cross validation showing the performance measures (prediction accuracy, *R*^2^, and *Q*^2^). *best values of *Q*^2^ (0.97). **(D)** The permutation test statistics (*p* < 5e^−4^). PLS score (*T*-score) explains *Y* and maximizes the relation between *X* and *Y*.

Substantial differences in the lipid profiles were observed in the mass spectra of the two groups (Figure 1). To evaluate these differences, the data were integrated and co-analyzed using unsupervised PCA (Figure S1 in Supplementary Material), supervised PLS-DA (Figure 2) and OPLS-DA methods (Figure S2 in Supplementary Material). Significant differences in the lipid profile were found between RAC patients and healthy subjects, with a satisfactory separation of the groups due to contrasting concentrations of constituents (Figure 2).

**Figure 3:**
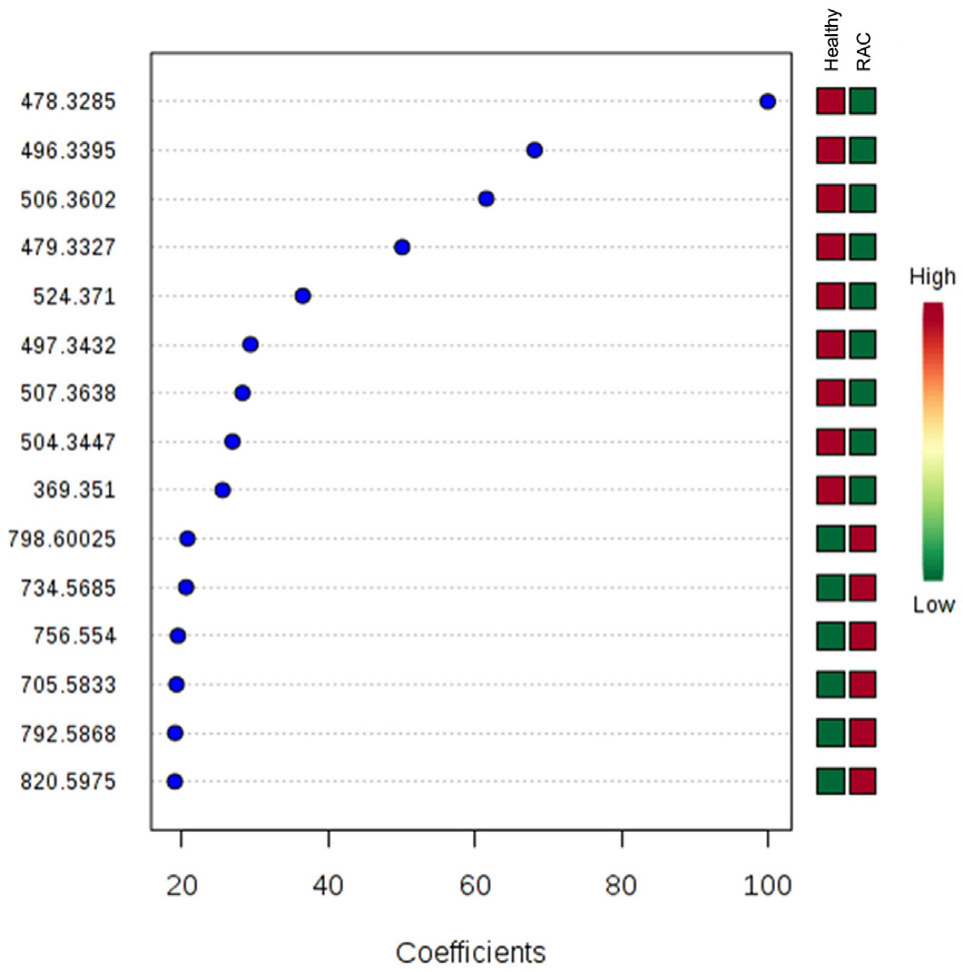
Important metabolite ions selected on the basis of variable importance in projection (VIP) score. The colored boxes on the right indicate relative bin integrals for healthy volunteers and rectal adenocarcinoma patients. VIP score is a weighted sum of squares of the PLS-DA loadings taking into account the amount of explained *Y*-variation in each dimension.

The index values of the variable importance in projection (VIP) from PLS-DA were used to measure the importance of each individual metabolite (Figure 3). A total of 120 variables were initially obtained and 15 ions (*m/z*) were highlighted by PLS-DA as important lipids to differentiate RAC patients from healthy volunteers. Nine of those ions were identified as shown in Table 2. Some of the ions had their identification confirmed by their collision-induced dissociation displayed in their MS/MS spectra (Table 2). Ions that could not be evaluated by MS/MS were identified with the SimLipid software (PREMIER Biosoft International, CA, USA).

**Table 2:** Main classes of lipids with contrasting abundances identified in the plasma of rectal cancer patients.

*m/z*	Lipid	Molecular formula	Lipid class	Tendency
734.5685^b^	Phosphatidylcholine (PC) (32:0)	C_40_H_80_NO_8_P	Glycerophosphocholine	High
	Phosphatidylethanolamine (PE) (35:0)		Glycerophosphoethanolamine	
756.554^b^	PC (31:3)	C_42_H_78_NO_8_P	Glycerophosphocholine	High
	PE (34:3)		Glycerophosphoethanolamine	
792.5868^b^	PC (P-38:5)	C_46_H_82_NO_7_P	Glycerophosphocholine	High
	PC (O-38:6)			
820.5975^b^	PC (P-40:5)	C_48_H_86_NO_7_P	Glycerophosphocholine	High
	PC (O-40:6)			
478.3285^a^	Lisophosphatidylcholine (LPC) (P-16:1)	C_24_H_48_NO_6_P	Glycerophosphocholine	Low
496.3395^a^	LPC (16:0)	C_24_H_50_NO_7_P	Glycerophosphocholine	Low
506.3602^a^	LPC (P-18:1)	C_26_H_52_NO_6_P	Glycerophosphocholine	Low
504.3447^a^	LPC (P-18:2)	C_26_H_50_NO_6_P	Glycerophosphocholine	Low
524.371^a^	LPC (18:0)	C_26_H_54_NO_7_P	Glycerophosphocholine	Low

*^a^Identified by MS/MS fragmentation pattern*.

*^b^Identified by exact mass only*.

Top 15 variables (ions with their *m/z*) that significantly contributed to the discrimination between groups were selected, resulting in a 20 VIP value cut-off. In the RAC group, ions of *m/z* 734 and 756 identified as PC or PE; and ions of *m/*z 792, 820 identified as PC plasmalogen (PCP) or 1-alkyl PC (PCO) were up-regulated, while lisophosphatidylcholine (LPC) metabolites (*m/z* 496 and 524) and three LPC plasmalogens (*m/z* 478, 506 and 504) were downregulated compared with healthy subjects. LPC plasmalogen of *m/z* 478 (LPC P-16:1) was the most relevant for predicting the response variable.

Table 3 sets out the results for the plasma FAs of the healthy volunteers and RAC patients in the different cancer stages. The main FAs found in all groups were the SFA followed by the *n −* 6 PUFAs and the MUFAs. No significant differences were observed in total MUFA and *n −* 6 PUFA amounts between the 0, I/II, and III/IV cancer stages, except for 16:1 *n −* 7 (*p* = 0.0455) and 20:4 *n −* 6 (*p* = 0.0472) which were reduced in the I/II cancer stages. Reductions were also observed in *n −* 3 PUFA, docosapentaenoic (C22:5 *n −* 3) (*p* = 0.0268) and docosahexaenoic (C22:6 *n −* 3) (*p* = 0.0086) acids in patients at the I/II cancer stages and docosapentaenoic (C22:5 *n −* 3) (*p* = 0.0330) acid was reduced in the III/IV cancer stages. The plasma of patients at stage 0 (carcinoma *in situ*) showed no alterations in FAs composition. As for the sum of the FAs classes (SFA, MUFA, and PUFA), there were no statistical differences among the groups evaluated.

**Table 3:** Fatty acid (FA) composition (% total relative) of plasma total lipids in healthy volunteers (*n* = 18) and rectal adenocarcinoma (RAC) patients in the different cancer stages.

Fatty acids	Healthy volunteers (*n* = 18)	Stage 0^a^ (*n* = 4)	Stages I/II (*n* = 9)	Stages III/IV (*n* = 10)
16:0	27.33 ± 4.37	26.27 ± 4.14	28.67 ± 2.75	28.96 ± 2.64
18:0	7.68 ± 1.44	8.11 ± 0.89	7.39 ± 0.96	7.48 ± 1.12
Σ SFA	35.01 ± 5.81	34.37 ± 5.03	36.07 ± 3.71	36.44 ± 3.76
16:1 *n −* 7	5.08 ± 0.79	4.21 ± 1.20	4.18 ± 1.45* (*p* = 0.0455)	4.25 ± 1.97
18:1 *n −* 9	20.06 ± 3.36	19.73 ± 4.28	20.25 ± 4.38	21.29 ± 1.92
Σ MUFA	25.14 ± 4.15	23.93 ± 5.48	24.23 ± 5.83	25.54 ± 3.90
18:2 *n −* 6	28.35 ± 5.66	27.08 ± 5.20	28.89 ± 5.97	25.82 ± 5.51
20:4 *n −* 6 (ARA)	5.12 ± 1.01	6.26 ± 2.42	4.33 ± 0.72* (*p* = 0.0472)	6.04 ± 2.50
Σ *n −* 6 PUFA	33.47 ± 6.67	33.33 ± 7.62	33.22 ± 6.69	31.85 ± 8.01
18:3 *n −* 3	1.35 ± 1.38	1.91 ± 1.36	0.97 ± 0.64	0.91 ± 0.59
20:5 *n −* 3 (EPA)	0.54 ± 0.42	0.60 ± 0.55	0.37 ± 0.23	0.30 ± 0.13
22:5 *n −* 3 (DPA)	0.33 ± 0.19	0.49 ± 0.43	0.17 ± 0.10* (*p* = 0.0268)	0.19 ± 0.06* (*p* = 0.0330)
22:6 *n −* 3 (DHA)	0.63 ± 0.28	0.79 ± 0.75	0.35 ± 0.12* (*p* = 0.0086)	0.49 ± 0.18
Σ *n −* 3 PUFA	2.85 ± 2.27	3.79 ± 3.08	1.86 ± 1.09	1.88 ± 0.97
Σ *n −* 6 PUFA/Σ *n −* 3 PUFA	11.7 ± 8.4	11.5 ± 7.4	17.8 ± 9.2	16.9 ± 9.5

*p < 0.05 compared to the healthy volunteers group (mean ± SD. Tukey test among three or more groups).

*^a^Patients in the stage 0 group were excluded*.

*SFA, saturated fatty acid; MUFA, monounsaturated fatty acid; PUFA, polyunsaturated fatty acid*.

### Lipid Peroxidation Determination

**Figure 4:**
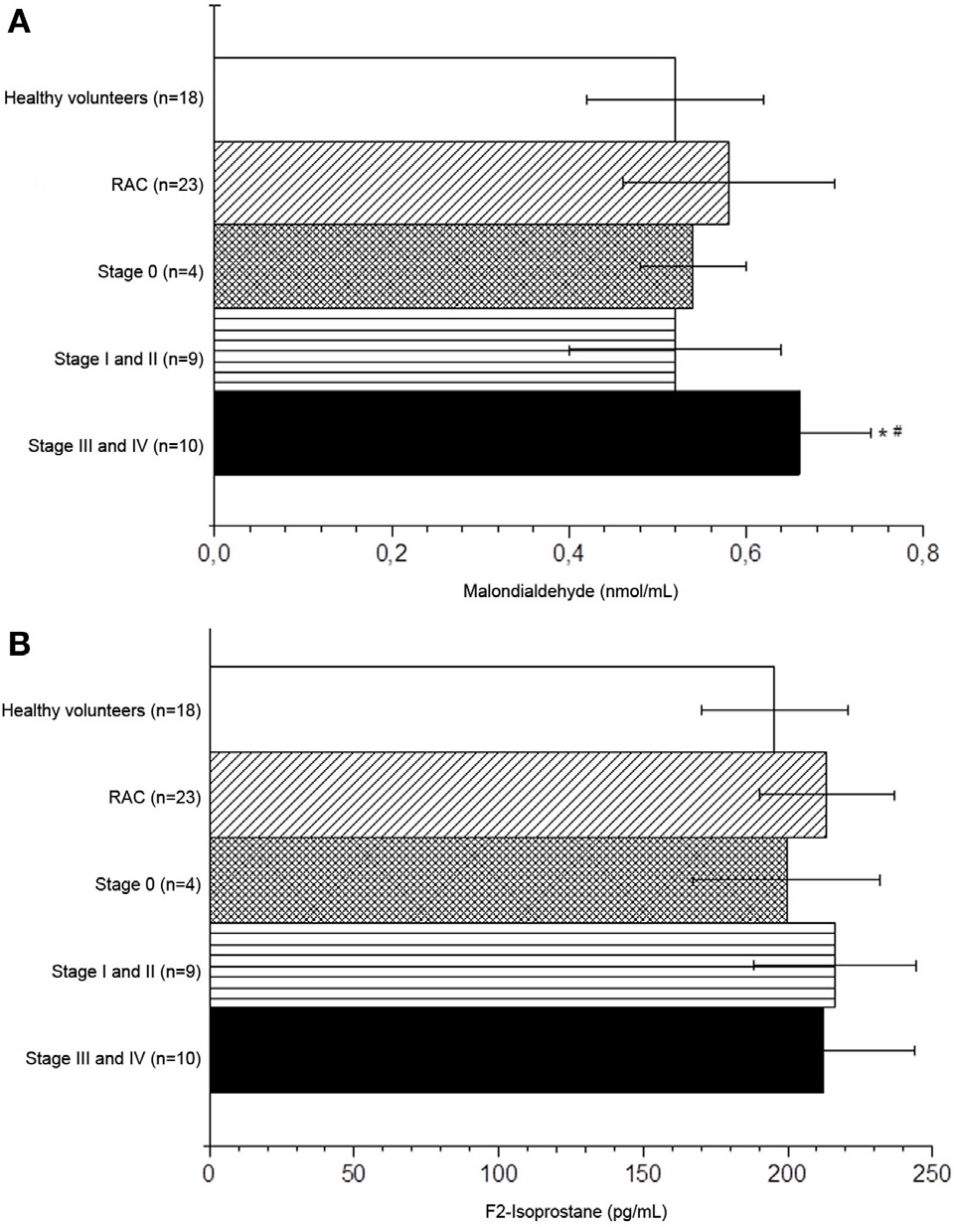
Malondialdehyde **(A)** and F_2_-isoprostane **(B)** levels of the healthy volunteers and rectal adenocarcinoma patients in the different cancer stages. *p < 0.05 compared to the healthy volunteers. ^#^*p* < 0.05 compared to stage 0; ANOVA followed by Tukey test. 1. Healthy volunteers, 2. Stages 0, 3. Stages I/II, 4. Stages III/IV.

The plasma levels of MDA (nmol/mL) and of F_2_-isoprostane (pg/mL) of healthy volunteers and the three groups separated by the staging are set out in Figures 4A,B, respectively. The plasma levels of MDA in the III/IV cancer stages were higher than the values observed in the healthy volunteers and in the stage 0 group (*p* < 0.05). No differences in MDA levels were observed in patients in the early stages (stages 0 and I/II) compared to healthy volunteers (Figure 4A). As regards the F_2_-isoprostane levels, there were no statistical differences among the groups evaluated (Figure 4B).

## Discussion

Our results show a significant difference in the lipid profile between RAC and healthy subjects. PC/PE and PCP were upregulated while those of LPC metabolites with SFAs (16:0 and 18:0) as well as unsaturated plasmalogens (16:1, 18:1, and 18:2) were significantly lower compared with healthy subjects. Also, a relative reduction of *n −* 3 PUFA, already observed in the early stage of cancer, and an increase of MDA, a biomarker of oxidative stress-induced lipid peroxidation, were observed in the plasma of these patients. The increases in MDA levels associated with high levels of plasma PCPs are a confirmation of the increased levels of oxidative stress.

Plasmalogens are a unique class of glycerophospholipids containing a fatty alcohol linked by a vinyl-ether moiety at the *sn −* 1 position of the glycerol backbone (29). It has been suggested that plasmalogens act as molecular scavengers (29), protecting cells and lipids against oxidative stress (30, 31), as their vinyl-ether bond is highly sensitive to oxidative attack compared to its ester counterparts. *In vitro* studies have indicated that plasmalogens are capable of reducing the oxidation of cell membrane PUFA and cholesterol (32, 33) and have also been correlated with the reduction in the levels of several oncogenic signaling lipids involved in the regulation of cell survival, cancer aggressiveness, and tumor growth (34). By contrast, LPC plasmalogens were decreasing in RAC patients; this may occur due to increased actions of lyso-PC acyltransferases (LPCAT). The exacerbated lipid anabolic metabolism in tumor cells is reflected in elevated activity and expression of several lipogenic enzymes. The LPCAT1, which converts LPC into PCs, is overexpressed in several cancers and is associated with colon cancer growth (21, 22) showed that LPCAT4 was overexpressed in CRC and contributes to PC (16:0/16:1) accumulation *via* the enhanced re-acylation of LPC.

Also, an abnormal choline-containing compounds metabolism have been identified and associated with oncogenesis and tumor progression (35). Choline kinase-α (Chk-α) activity, an enzyme in the Kennedy pathway that phosphorylates free choline to PC, and PC levels were increased in colon cancer and adenoma tissue (36).

The existence of LPC P16:1 was, however, reported here for the first time in the plasma of patients with RAC. The presence of a plasmalogen in primary and metastatic CRC cell lines (37) and in exosomes secreted by the CRC cell line (23) has been reported previously. The functional role of changes in plasmanyl-lipids in malignancy and metastasis are still poorly understood. It is unclear whether decreased levels of LPC plasmalogens are due to metabolic alteration in RAC or whether plasmalogens play an important role in the pathogenesis of RAC. High levels of PC (C30:0) and low levels of LPC (C18:0) were consistently related to higher risks of colorectal, breast, and prostate cancer, independently of background factors, such as age, BMI, dietary factors, or smoking status (38). In our study, the lipidomic signature was not correlated with demographic, anthropometric, clinical, or other lifestyle factors given that the number of subjects was insufficient for that to be done.

Our results show a relative reduction of MUFA (16:1), *n −* 6 PUFA (20:4), and *n −* 3 PUFA (22:5 and 22:6) in plasma of early stages CRC patients. The observation of downregulation in FAs during cancer progression is in accordance with the previous studies of the hypermetabolic response and increased energy expenditure of cancer patients (39). Preliminary data reported a higher proportion of palmitic acid (18:0) and a lower eicosapentaenoic (20:5 *n −* 3) proportion in the plasma PL fraction in the CRC patients than in the controls (40). A decrease of the 16:1 *n −* 7/16:0 and 18:1 *n −* 9/18:0 ratios in colorectal tissue cancer has previously been observed, suggesting that change in FA ratios may be involved in the mechanism of CRC progression (41). Li et al. (42) reported that the reduction of oleamide and FAs (16:2, 18:0, and 20:3) in serum achieved excellent diagnostic accuracy for differentiating early stages CRC patients from healthy controls. Although we were not able to identify differences in oxidative stress as estimated by 8-isoprostane levels between groups, we found that patients with RAC at stages III and IV have increased MDA values. The relation between the staging of the tumor and the increase in oxidative stress remains unclear. Previous research has shown that higher levels of oxidative stress markers are associated with an advanced state of cancer. Mendonça et al. (43) reported that higher MDA levels were found in the serum of patients with CRC at an advanced stage (III). Surinenaite et al. (44) also reported that in 65 patients serum MDA level was more elevated in advanced than early stage CRC. The serum MDA level was significantly decreased after surgical treatments compared to pre-surgical status. Gopčević et al (45) showed, however, that Japanese patients with CRC had high plasmatic levels of MDA at all stages compared to healthy individuals and there were no differences among the stages of the disease.

## Conclusion

Early stage RAC was characterized *via* MS lipid profiles by a reduced level of *n −* 3 PUFA and LPC plasmalogen species. Our results pointed to this class as a disease biomarker and a better oxidative stress evaluator, since plasmalogen alterations can be observed in early stages of RAC. Plasmalogens might, therefore, work as biomarkers for diagnosis and also be an index for the surveillance of treatment effects. This was an explorative study and the observed associations need to be validated in a larger cohort that would make it feasible to adjust for more potential confounders in multivariate statistical tests, nevertheless, the results are indeed promising.

## Ethics Statement

The project was approved by the ethics committee of the São Francisco University (CAAE: 51356315.5.0000.5514) and by the Faculty of Medical Sciences of the State University of Campinas (CAAE: 08287012.0.0000.5404).

## Author Contributions

PC, MM, and GM: conception and design of research; GM, LC, CM, and CC: acquisition of samples; MM and CA: performed experiments; MM, CA, and ME: acquisition of data; PC, MM, and CA: analyzed data and interpreted results of experiments; PC, MM, and CA: drafted the manuscript; and PC, MM, GM, CA, ME, CM, and CC: revised and approved final version of manuscript.

## Conflict of Interest Statement

The authors declare that the research was conducted in the absence of any commercial or financial relationships that could be construed as a potential conflict of interest. The reviewer UM and handling editor declared their shared affiliation.
