# Supplementary material for: Plasma Lipidomic Signature of Rectal Adenocarcinoma Reveals Potential Biomarkers
Source: Front Oncol. 2018 Jan 8;7:325. doi: 10.3389/fonc.2017.00325 (PMC5766651; doi:10.3389/fonc.2017.00325)

**Supplementary Figures**

**
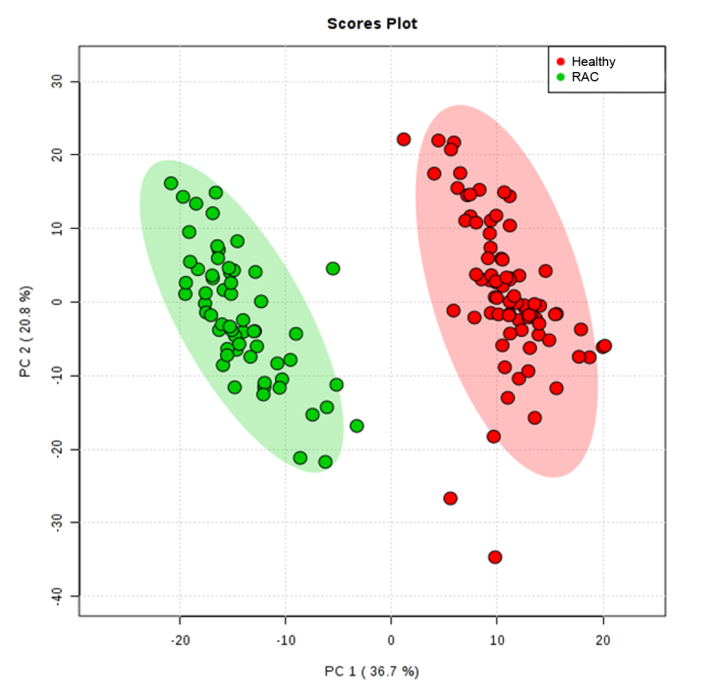

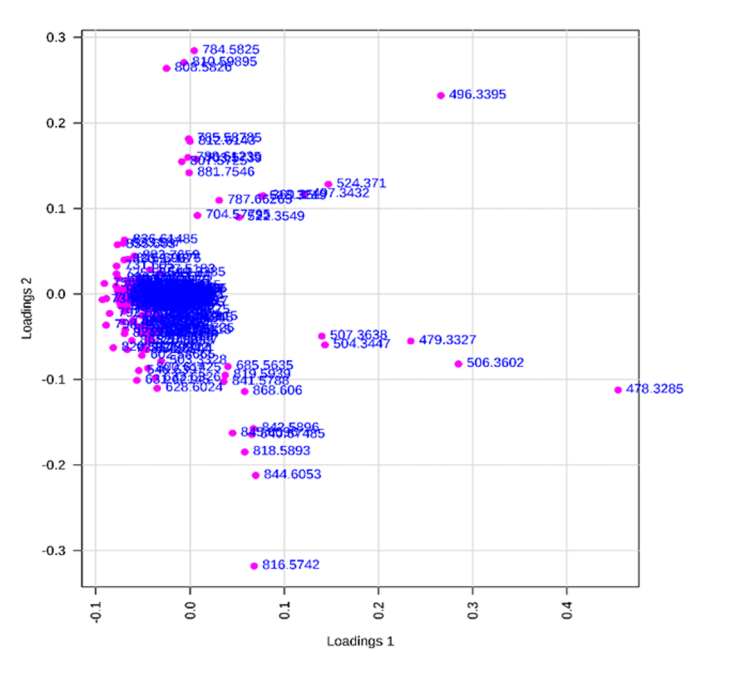
**

**B**

**A**

**Figure S1:** (A) PCA scores plot of mass spectra data of human plasma extract from healthy volunteers (red) and RAC patients (green). (B) Loadings plot for PC1 and PC2 showing the important metabolites (m/z).


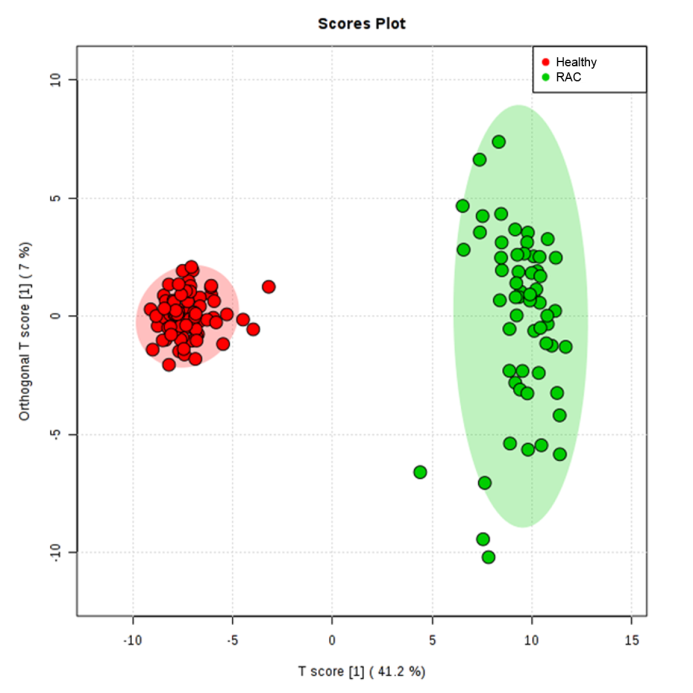

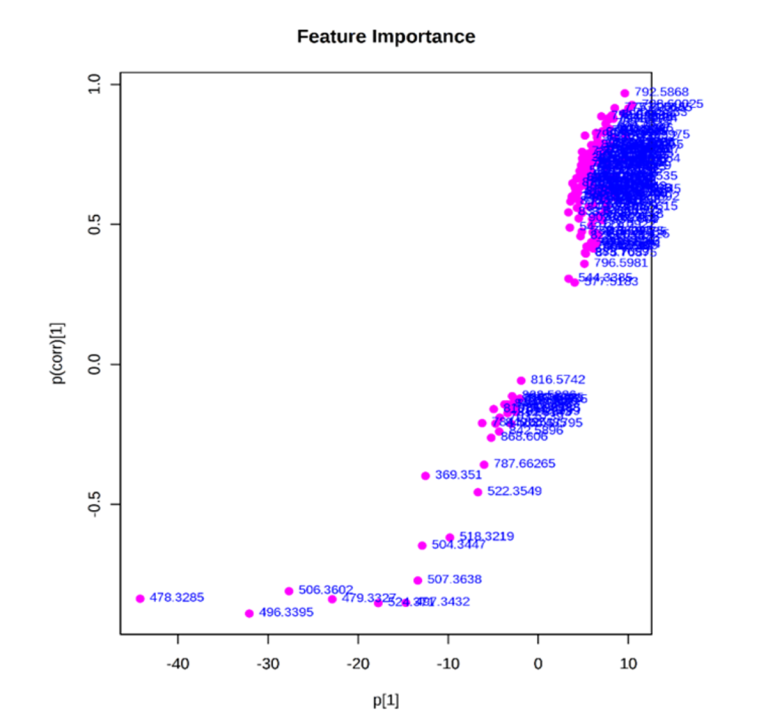


**B**

**A**

**Figure S2:** (A) OPLS-DA scores plot of healthy volunteers (red) and RAC patients (green). 41.2% and 7% are the scores of the T score and orthogonal T score, respectively. (B) Loadings plot showing the important metabolites (m/z) for the group discrimination.

**Ethics committee of the São Francisco University**


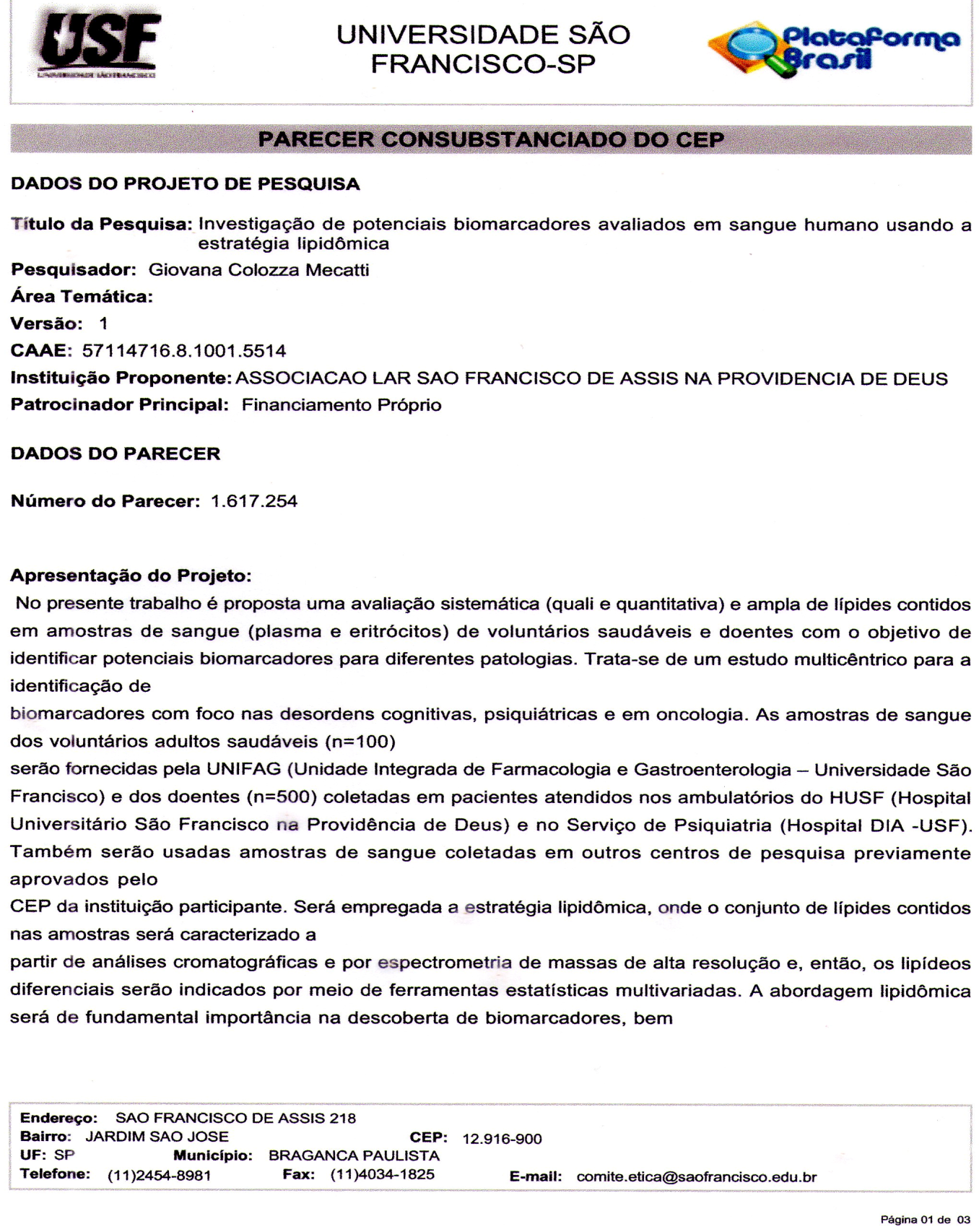


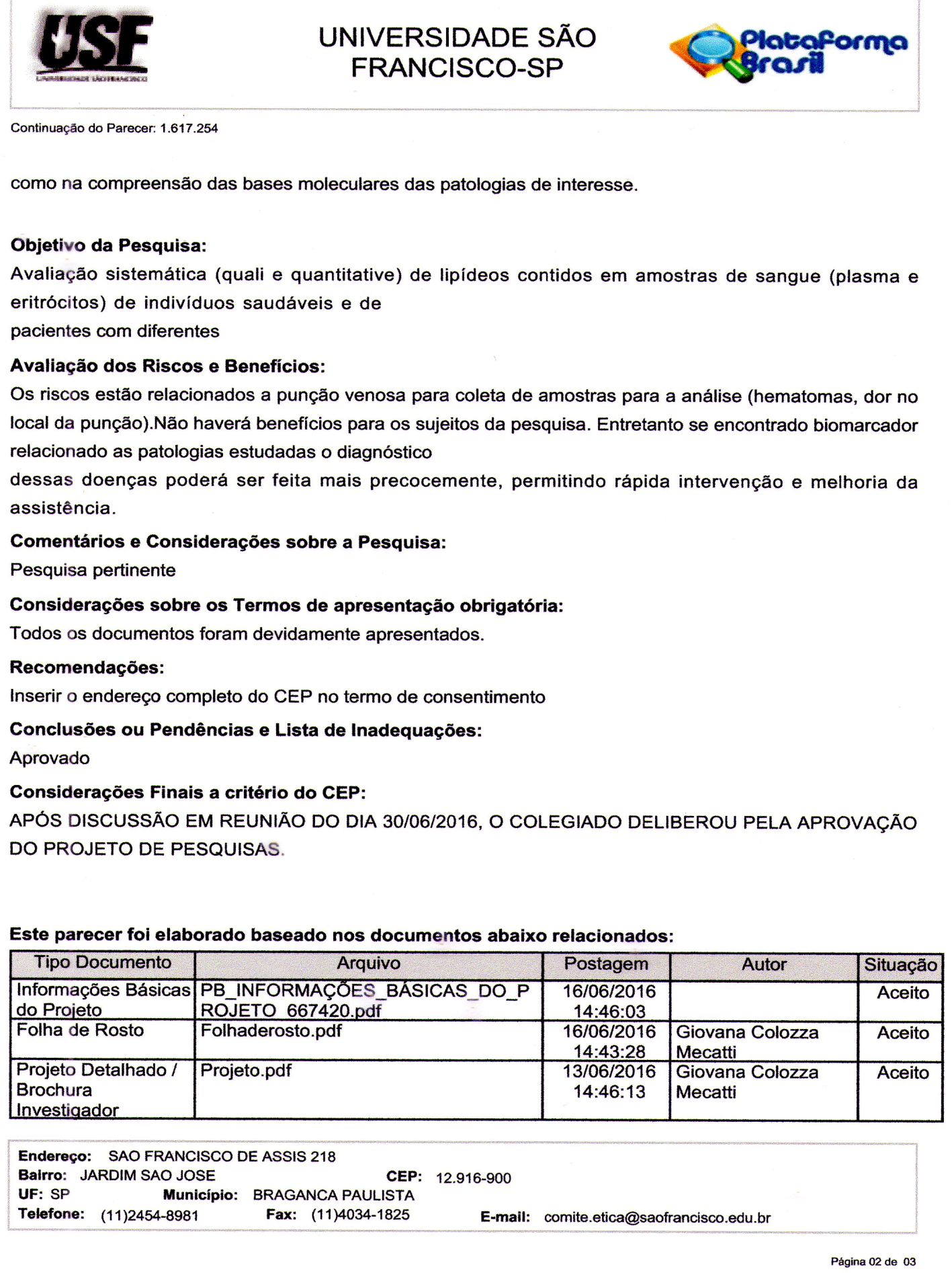

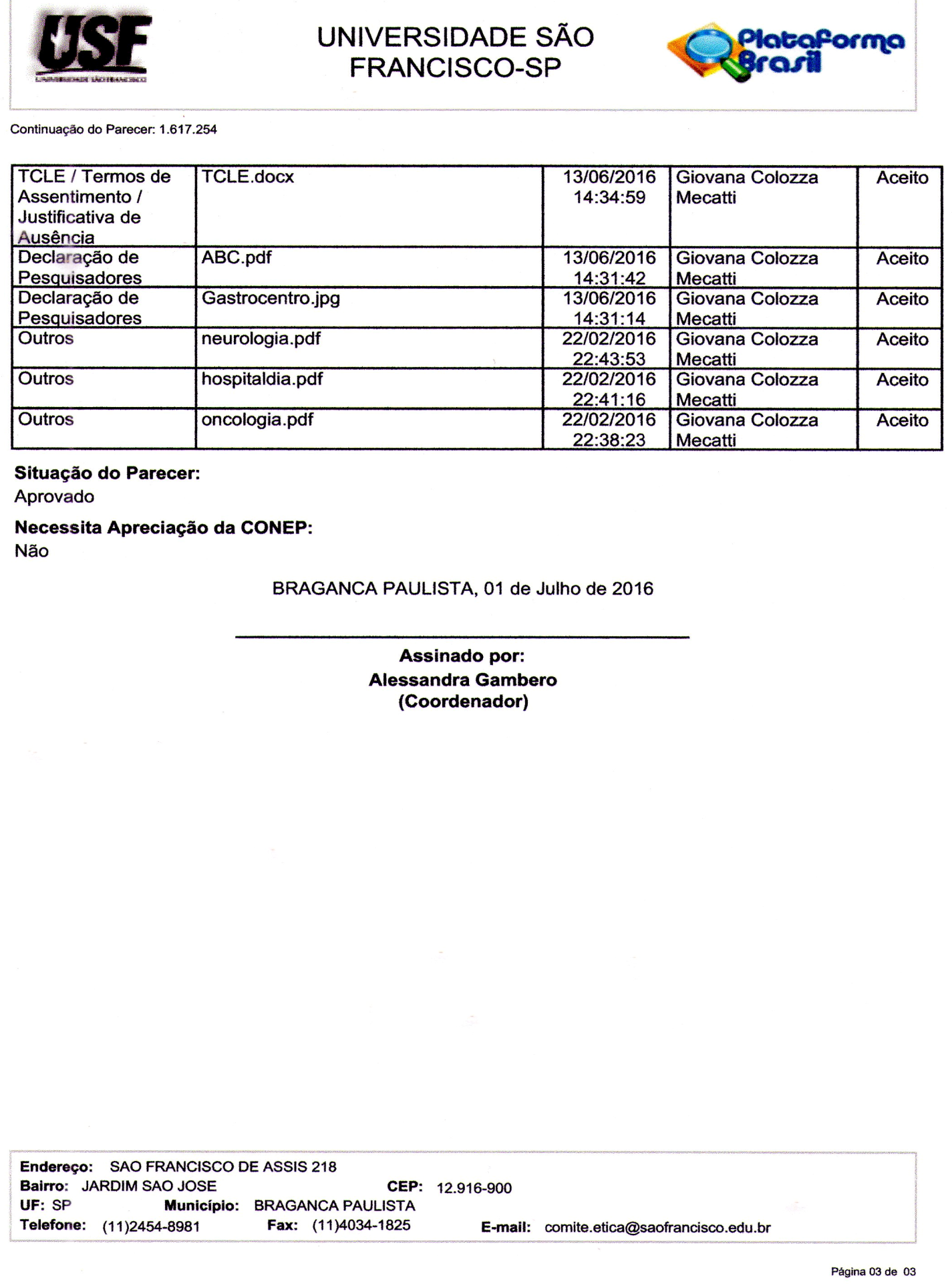


**Ethics committee of the Faculty of Medical Sciences of the State University of Campinas**


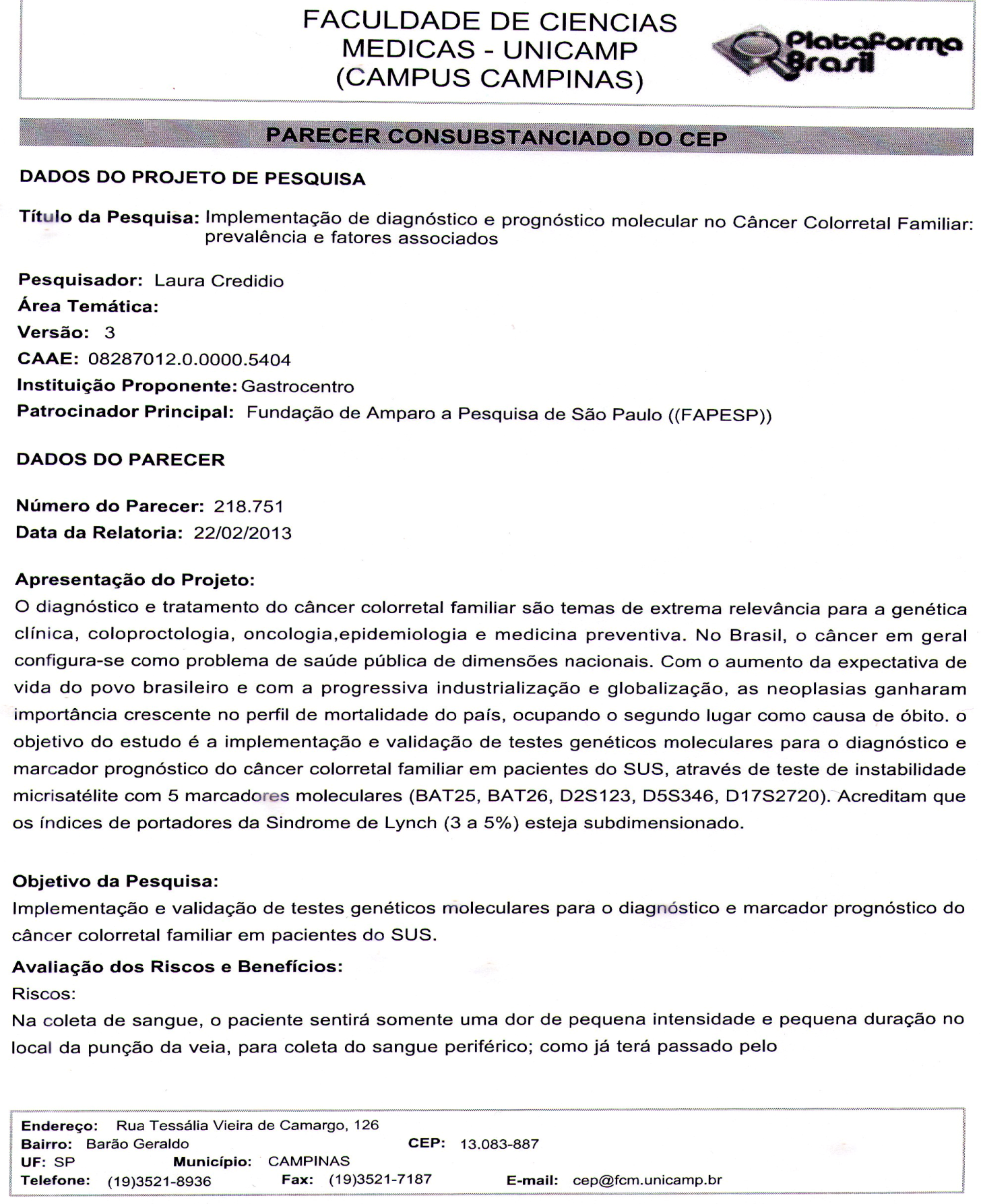


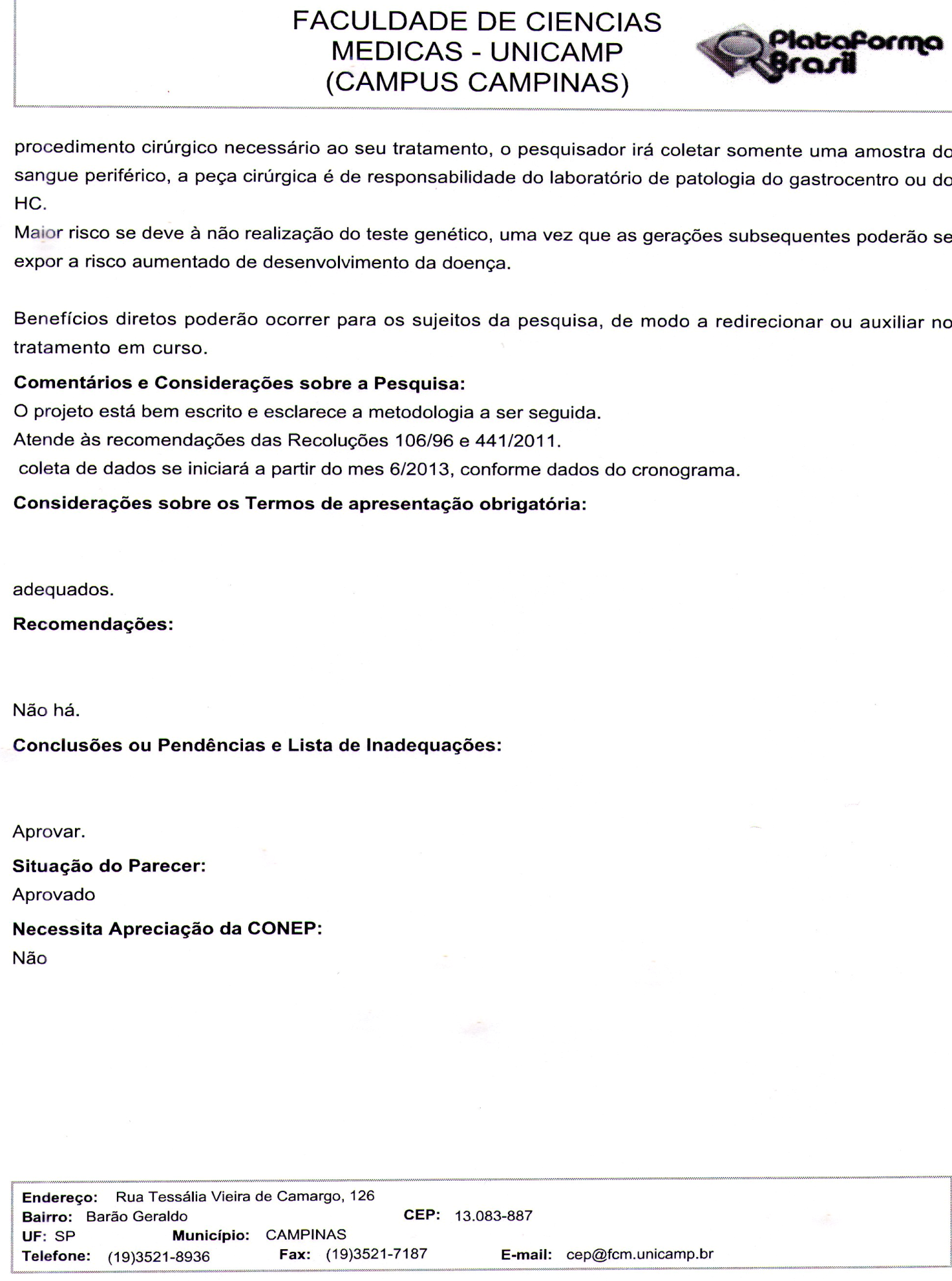

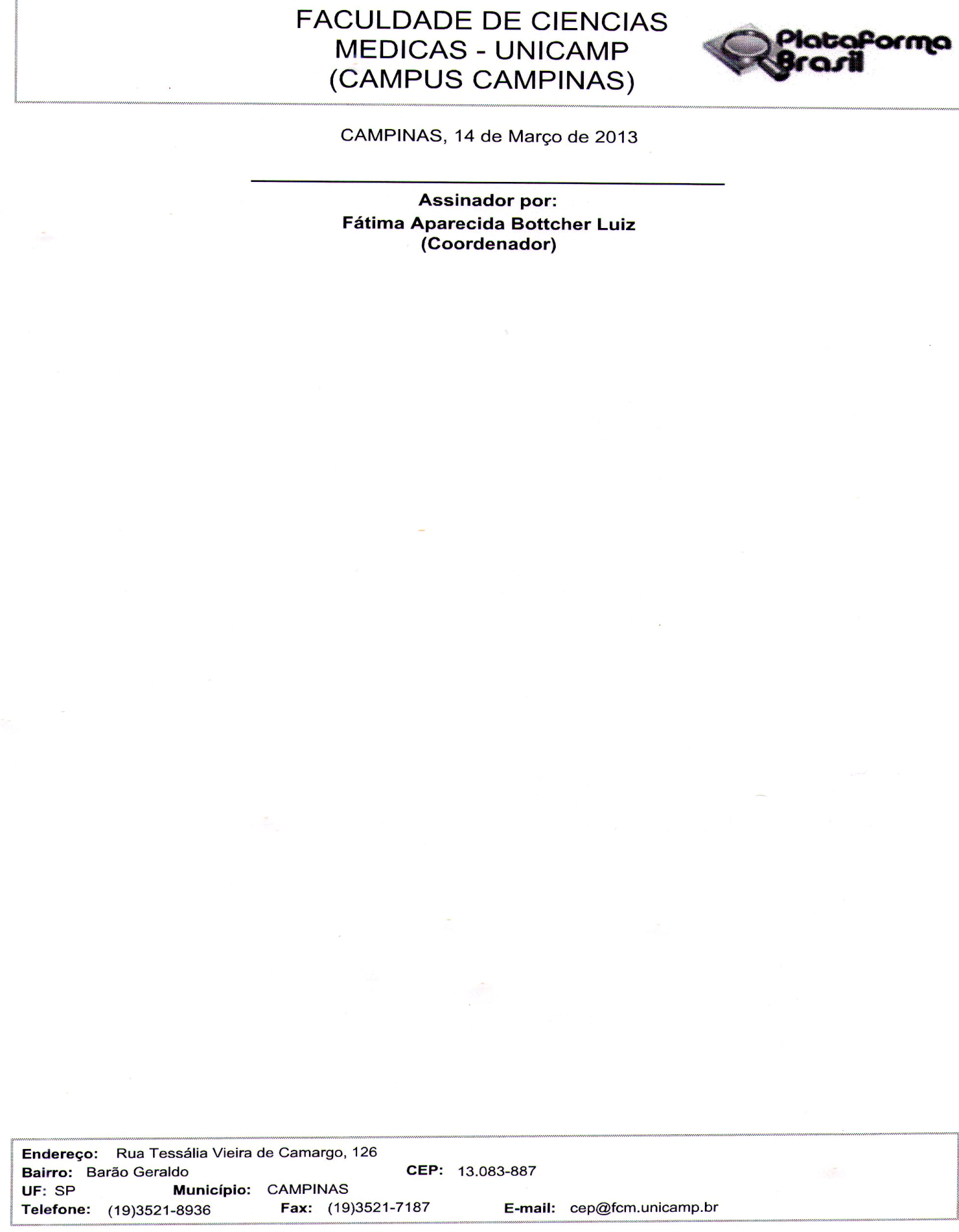

Supplement: Supplementary file 1 [file Data_Sheet_1.docx]
